# A Multicentre Study of 5-year Outcomes Following Focal Therapy in Treating Clinically Significant Nonmetastatic Prostate Cancer

**DOI:** 10.1016/j.eururo.2018.06.006

**Published:** 2018-10

**Authors:** Stephanie Guillaumier, Max Peters, Manit Arya, Naveed Afzal, Susan Charman, Tim Dudderidge, Feargus Hosking-Jervis, Richard G. Hindley, Henry Lewi, Neil McCartan, Caroline M. Moore, Raj Nigam, Chris Ogden, Raj Persad, Karishma Shah, Jan van der Meulen, Jaspal Virdi, Mathias Winkler, Mark Emberton, Hashim U. Ahmed

**Affiliations:** a Division of Surgery and Interventional Sciences, University College London, London, UK; b Department of Urology, UCLH NHS Foundation Trust, London, UK; c Department of Radiotherapy, University Medical Centre, Utrecht, The Netherlands; d Imperial Urology, Charing Cross Hospital, Imperial College Healthcare NHS Trust, London, UK; e Department of Urology, The Princess Alexandra Hospital NHS Trust, Harlow, UK; f Department of Urology, Dorset County Hospital NHS Trust, Dorset, UK; g Department of Urology, University Hospital Southampton NHS Trust, Southampton, UK; h Division of Surgery, Department of Surgery and Cancer, Faculty of Medicine, Imperial College London, London, UK; i Department of Urology, Basingstoke and North Hampshire Hospital, Hampshire Hospitals NHS Foundation Trust, Basingstoke, UK; j Springfield Hospital, Chelmsford, UK; k Department of Urology, Royal County Surrey Hospital NHS Trust, Guildford, UK; l Department of Academic Urology, The Royal Marsden Hospital NHS Foundation Trust, London, UK; m Department of Urology, Southmead Hospital, North Bristol NHS Trust, Bristol, UK; n London School of Hygiene and Tropical Medicine, London, UK

**Keywords:** High-intensity focused ultrasound, Focal therapy, Transperineal biopsy, Multiparametric magnetic resonance imaging, Targeted biopsy

## Abstract

**Background:**

Clinically significant nonmetastatic prostate cancer (PCa) is currently treated using whole-gland therapy. This approach is effective but can have urinary, sexual, and rectal side effects.

**Objective:**

To report on 5-yr PCa control following focal high-intensity focused ultrasound (HIFU) therapy to treat individual areas of cancer within the prostate.

**Design, setting, and participants:**

This was a prospective study of 625 consecutive patients with nonmetastatic clinically significant PCa undergoing focal HIFU therapy (Sonablate) in secondary care centres between January 1, 2006 and December 31, 2015. A minimum of 6-mo follow-up was available for599 patients. Intermediate- or high-risk PCa was found in 505 patients (84%).

**Intervention:**

Disease was localised using multiparametric magnetic resonance imaging (mpMRI) combined with targeted and systematic biopsies, or transperineal mapping biopsies. Areas of significant disease were treated. Follow-up included prostate-specific antigen (PSA) measurement, mpMRI, and biopsies.

**Outcome measurements and statistical analysis:**

The primary endpoint, failure-free survival (FFS), was defined as freedom from radical or systemic therapy, metastases, and cancer-specific mortality.

**Results and limitations:**

The median follow-up was 56 mo (interquartile range [IQR] 35–70). The median age was 65 yr (IQR 61–71) and median preoperative PSA was 7.2 ng/ml (IQR 5.2–10.0). FFS was 99% (95% confidence interval [CI] 98–100%) at 1 yr, 92% (95% CI 90–95%) at 3 yr, and 88% (95% 85–91%) at 5 yr. For the whole patient cohort, metastasis-free, cancer-specific, and overall survival at 5 yr was 98% (95% CI 97–99%), 100%, and 99% (95% CI 97–100%), respectively. Among patients who returned validated questionnaires, 241/247 (98%) achieved complete pad-free urinary continence and none required more than 1 pad/d. Limitations include the lack of long-term follow-up.

**Conclusions:**

Focal therapy for select patients with clinically significant nonmetastatic prostate cancer is effective in the medium term and has a low probability of side effects.

**Patient summary:**

In this multicentre study of 625 patients undergoing focal therapy using high-intensity focused ultrasound (HIFU), failure-free survival, metastasis-free survival, cancer-specific survival, and overall survival were 88%, 98%, 100%, and 99%, respectively. Urinary incontinence (any pad use) was 2%. Focal HIFU therapy for patients with clinically significant prostate cancer that has not spread has a low probability of side effects and is effective at 5 yr.

## Take Home Message

Our study shows that at median follow-up of approximately 5 yr after focal high-intensity focused ultrasound, nine out of ten patients with predominantly intermediate- or high-risk prostate cancer are able to avoid surgery, radiotherapy, and systemic therapy. Prostate cancer–specific survival was 100%. There was a low probability of urinary incontinence (2%), erectile dysfunction (15%), and rectal side effects (rare).

## 1 Introduction

The therapeutic approach to nonmetastatic prostate cancer is an outlier compared to strategies for other solid organ cancers. Regardless of the burden or location of cancer within the gland, treatment is directed at the whole gland using surgery or radiotherapy. While both approaches are effective, they can be associated with urinary incontinence and erectile dysfunction, with radiotherapy occasionally causing rectal side effects [1], [2], [3]. While current trends demonstrate that radical therapy is increasingly used among the patients most likely to benefit from treatment with respect to better cancer-specific survival [4], there is still a need to reduce treatment-related side effects, particularly as the survival benefit conferred by radical treatment is often seen over 10–15 yr when compared to a strategy of active monitoring [5].

The aim of focal therapy is to reduce side effects and maintain cancer control by targeting areas of known cancer in a similar approach to that for other solid organ cancers [6], [7], [8]. This concept of tissue preservation has come about through improvements in disease localisation using multiparametric magnetic resonance imaging (mpMRI) and mapping biopsy techniques [9]. However, many studies have hitherto been small, based in expert centres, or subject to short follow-up [10], [11], [12], [13], [14], [15], [16], [17], [18]. The only randomised study to evaluate focal therapy recruited patients with low-risk disease [19], a population known to have little to no chance of metastases or cancer-related mortality.

We now report on medium-term cancer control outcomes in a large multicentre patient cohort with clinically significant nonmetastatic prostate cancer treated with focal therapy using high-intensity focused ultrasound (HIFU).

## 2 Patients and methods

Institutional review board exemption was granted by University College London Hospital for our health technology assessment programme, which followed guidelines for evaluating surgical interventions [20], [21]. Focal HIFU commenced in 2006 in the UK with approval for clinical use by the National Institute for Health and Care Excellence (NICE) under special arrangements. All cases had to be prospectively and consecutively entered into an academic registry, discussed in a multidisciplinary meeting, and given written information on the advantages and disadvantages of the procedure. We previously reported on medium-term outcomes following whole-gland HIFU [22]. Between January 1, 2006 and December 31, 2015, 625 consecutive patients underwent primary focal HIFU using a Sonablate 500 device (Sonacare Inc., Charlotte, NC, USA) in nine centres. Focal HIFU treatment was offered to patients diagnosed with nonmetastatic prostate cancer of Gleason score 6–9 and stage T1c–3bN0M0 and prostate-specific antigen (PSA) of ≤30 ng/ml. Gleason 6 disease required a minimum of 4 mm of cancer [23]. Patients were classified into D’Amico low-, intermediate-, and high-risk groups [24].

Disease was localised using mpMRI combined with targeted and systematic biopsies, or transperineal mapping biopsies. Targeted biopsies involved taking three to six biopsy cores from all lesions with a Likert score of 3–5, as well as systematic biopsies (transperineal or transrectal). Mapping biopsies involved taking biopsy cores every 5 mm. Intermediate- and high-risk cases also underwent a radioisotope bone scan and/or cross-sectional computed tomography to rule out distant metastases, depending on local institution guidelines.

All surgeons underwent a rigorous period of training involving online learning, observation of five cases on two separate occasions in an expert centre, and on-site proctoring by an expert urologist for five to ten cases, and were followed by an expert clinical applications specialist for all subsequent treatments.

**Fig. 1:**
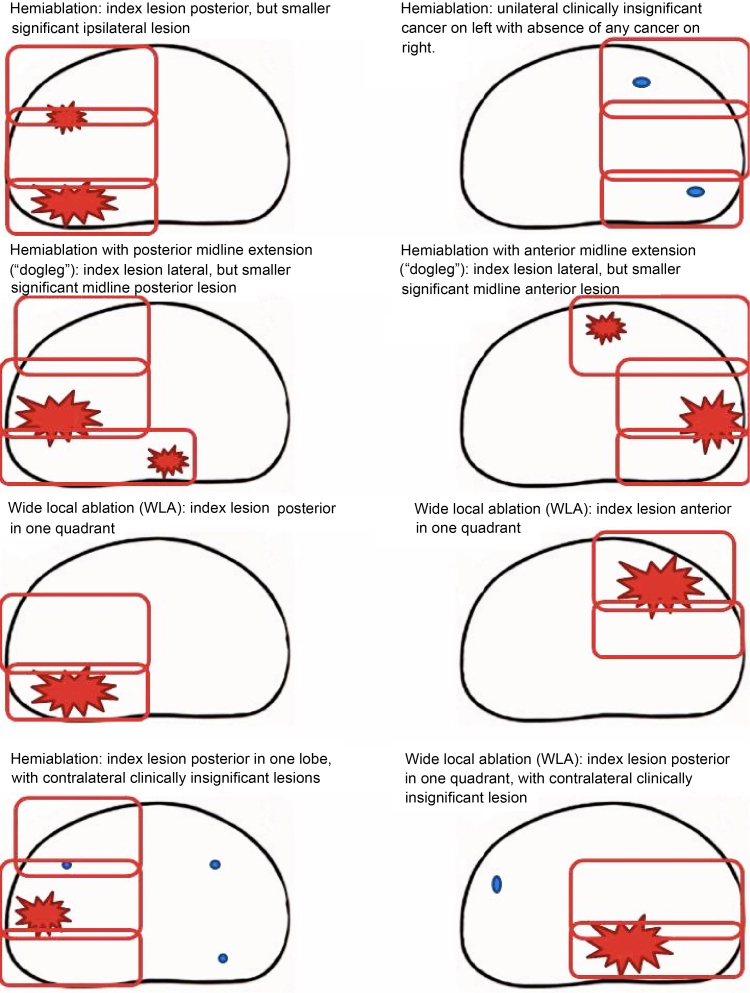
Types of focal therapy using high-intensity focused ultrasound (HIFU) carried out in men with non-metastatic prostate cancer.

There were various forms of focal HIFU permitted (Fig. 1). Up to two retreatments with focal HIFU were allowed. All men were advised to undergo 3–6-monthly PSA testing, with mpMRI routinely performed at 1 yr and every 1–2 yr. Two rises in PSA after the nadir, without predefining the magnitude of the rise, were investigated using prostate biopsy or mpMRI followed by biopsy if the mpMRI was suspicious. We have previously reported on the high negative predictive value of mpMRI following focal-HIFU [23], [25].

Repeat HIFU was offered when either (1) clinically significant cancer on biopsy occurred in field or out of field and mpMRI staging indicated that the disease was still localised, or (2) when mpMRI demonstrated clear in-field recurrence (mpMRI Likert score 5) associated with a rising PSA. Patients were routinely offered the option of radical prostatectomy or radical radiotherapy.

Physicians assessed postoperative adverse events during follow-up clinic visits. Functional outcomes were assessed via patient-reported outcome measures using validated questionnaires collected at 1–2 and 2–3 yr after focal HIFU treatment. Validated questionnaires included the International Prostate Symptom Score [26] and the Expanded Prostate Cancer Index Composite (EPIC) urinary continence domain [27]. All data were audited and quality controlled by two data managers (N.M. and F.H.J.).

### 2.1 Primary outcome

Failure-free survival (FFS) was defined as avoidance of local salvage therapy (surgery or radiotherapy), systemic therapy, metastases, and prostate cancer–specific death. This excluded PSA kinetics, as there are no kinetic measures that are valid in this setting.

### 2.2 Secondary outcomes

Our main secondary outcomes included metastasis-free survival and prostate cancer–specific and overall mortality. We also report biopsy outcomes when carried out, as well as adverse events and side effects. Urinary continence was defined as being completely pad-free and socially continent (0–1 pads/d). The latter definition is commonly used in many series reporting radical prostatectomy outcomes [28]. We also report on complete pad-free, leak-free urinary continence. In addition, we evaluated whether certain baseline factors might predict for FFS.

### 2.3 Statistical analysis

As this was a cohort registry study, there were no a priori sample size calculations. Our decision to analyse and publish these data was based on the registry being open for 10 yr and the availability of median follow-up of approximately 5 yr for the cohort. Baseline characteristics are presented as the median (interquartile range [IQR]) or proportion, as appropriate. Kaplan-Meier estimates of time-to-event outcomes are described with 95% confidence interval (CI) for those men with at least 6-mo follow-up. Adverse events are reported as proportions only, and for these the entire cohort was included to reduce selection bias. Urinary continence was evaluated using the EPIC urinary continence domain with a pad-free definition and a socially continent definition (0–1 pads).

Cox regression was used to determine whether baseline factors could predict FFS. The univariable analysis included age, prostate volume, PSA, Gleason score, and clinical T stage. For multivariable analysis, significant factors (*p* < 0.05) were included (Supplementary Table 1). R version 3.4.2 was used for all statistical analyses. (R Foundation for Statistical Computing, Vienna, Austria; www.R-project.org) The discriminative ability of the model was measured using Harrell's *c* statistic. The model was recalculated using 2000 bootstrap resamples to account for optimism, after which the *c* statistic was adjusted. The *c* statistic is comparable to the area under the receiver operating characteristic curve for survival models.

## 3 Results

### 3.1 Baseline demographics

A total of 625 patients were treated with focal HIFU, of whom 599 reached at least 6-mo follow-up and 505 (84%) had intermediate- or high-risk prostate cancer (Table 1). Table 2 lists the biopsy characteristics. When excluding patients with an event (*n* = 60) the median follow-up was 56 mo (IQR 35–70).

**Table 1:** Baseline characteristics for patients undergoing focal HIFU for nonmetastatic prostate cancer a

Characteristic	Group 1 (*n* = 599)
Median age, yr (IQR)	65 (61–71)
Median pre-HIFU PSA, ng/ml (IQR)	7.2 (5.2–10.0)
Missing data, *n* (%)	12 (2)
Pre-HIFU PSA group, *n* (%)	
<10 ng/ml	440 (75)
10–20 ng/ml	134 (23)
>20 ng/ml	13 (2)
Missing data	12 (2)
Median pre-HIFU prostate volume, ml (IQR)	37 (28–47)
Missing data, *n* (%)	37 (6.2)
Median pre-HIFU PSA density, ng/ml/ml (IQR)	0.19 (0.12–0.3)
Missing data, *n* (%)	46 (7.7)
Gleason score, *n* (%)	
3 + 3 = 6	166 (28)
3 + 4 = 7	327 (55)
4 + 3 = 7	86 (14)
≥8	11 (2)
Missing data	9 (1.5)
Pre-HIFU T stage, *n* (%)	
T1	65 (11)
T2	432 (72)
T2a	82 (14)
T2b	73 (12)
T2c	93 (16)
Missing T2 subclassification	184 (31)
T3a/b	75 (13)/7 (1.2)
Missing data	20 (3.3)
D’Amico risk group, *n* (%)	
Low	78 (13)
Intermediate	316 (53)
High	189 (32)
Missing data	16 (2.7)

HIFU = high-intensity focused ultrasound; IQR = interquartile range; PSA = prostate-specific antigen.

a Some cumulative percentages are higher than 100% because of rounding.

**Table 2:** Pre-HIFU biopsies

Type of biopsy/detection	Patients, *n* (%)	Median number of cores, *n* (IQR)	
		All cores	Positive cores
Transperineal mapping	420 (70)	37 (28–54) a	6 (4–10) c
Transurethral ultrasound	169 (28)	12 (10–13) b	4 (2–5) a
Transurethral resection of the prostate	1 (0.2)	NA	NA
Magnetic resonance imaging only	1 (0.2)	NA	NA
Unknown	1 (0.2)	NA	NA
Missing	7 (1.2)	NA	NA

HIFU = high-intensity focused ultrasound; IQR = interquartile range; NA = not applicable/available.

a Data missing for six patients.

b Data missing for 12 patients.

c Data missing for eight patients.

### 3.2 Primary outcome

**Fig. 2:**
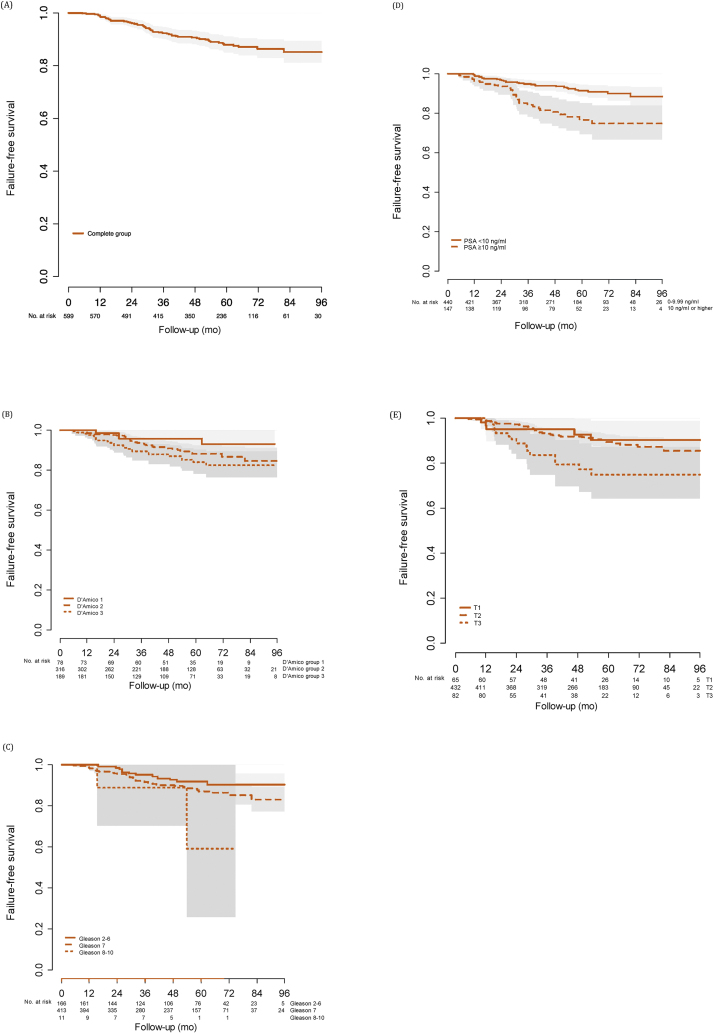
Kaplan-Meier curves showing failure-free survival for (A) the entire group, (B) by D’Amico risk group, (C) by Gleason score, (D) by prostate-specific antigen (PSA) category, and (E) by T stage.

The FFS at 1, 3, and 5 yr was 99% (95% CI 98–100%), 92% (95% CI 90–95%), and 88% (95% CI 85–91%), respectively (Table 3). Kaplan-Meier estimates at 5 yr were 96% (95% CI 91–100%), 88% (95% CI 84–93%), and 84% (95% CI 78–90%) for low-, intermediate. and high-risk groups, respectively (Table 3 and Fig. 2).

**Table 3:** Kaplan-Meier estimates of freedom from repeat HIFU, overall survival, metastasis-free survival, and overall failure-free survival following focal HIFU therapy among men treated for nonmetastatic prostate cancer

	Kaplan-Meier estimate, % (95% confidence interval)		
	1 yr	3 yr	5 yr
Overall survival	100 (99–100)	99 (98–100)	99 (97–100)
By D’Amico risk class			
Low	99 (96–100)	99 (96–100)	99 (96–100)
Intermediate	100 (99–100)	99 (98–100)	99 (97–100)
High	99.5 (98–100)	99 (97–100)	98 (96–100)
Metastasis-free survival	99.7 (99–100)	99 (98–100)	98 (97–99)
By D’Amico risk class			
Low	100 (NA)	99 (96–100)	96 (93–100)
Intermediate	99.7 (99–100)	99 (97–100)	99 (97–100)
High	99.5 (98–100)	98 (96–100)	97 (95–100)
Failure-free survival	99 (98–100)	92 (90–95)	88 (85–91)
By D’Amico risk class			
Low	99 (96–100)	96 (91–100)	96 (91–100)
Intermediate	99 (97–100)	93 (90–96)	88 (84–93)
High	98 (97–100)	89 (85–94)	84 (78–90)
By Gleason score			
≤6	99 (98–100)	95 (92–99)	92 (87–97)
7	99 (98–100)	92 (89–95)	87 (83–91)
≥8	89 (71–100)	89 (79–100)	59 (26–100)
By pre-HIFU PSA group			
<10 ng/ml	99.5 (99–100)	95 (93–97)	92 (89–95)
≥10 ng/ml	97 (94–100)	85 (78–91)	77 (69–84)
Free from repeat HIFU	98 (96–99)	84 (81–87)	75 (71–80)
By D’Amico risk class			
Low	97 (94–100)	82 (74–92)	78 (69–89)
Intermediate	97 (95–99)	88 (85–92)	79 (74–85)
High	98 (97–100)	76 (69–83)	68 (61–76)

HIFU = high-intensity focused ultrasound; NA = not applicable; PSA = prostate-specific antigen.

### 3.3 Secondary outcomes

Following focal HIFU, eight patients transitioned to salvage radical prostatectomy, 36 had salvage external beam radiotherapy, and one received androgen deprivation therapy. Ten patients experienced metastases during the follow-up, of whom two had low-risk, four had intermediate-risk, and four had high-risk disease; three had metastases after the second HIFU treatment. Kaplan-Meier estimates for metastasis-free survival at 1, 3, and 5 yr were 99.7% (95% CI 99–100%), 99% (95% CI 98–100%), and 98% (95% CI 97–99%), respectively (Table 3). There were seven deaths, none of which was related to prostate cancer. Kaplan-Meier estimates for overall survival at 1, 3, and 5 yr were 100% (95% CI 99–100%), 99% (95% CI 98–100%), and 99% (95% CI 97–100%), respectively. At least one repeat focal HIFU treatment was performed in 121 patients (one repeat HIFU in 112 and two repeat HIFU in 9).

A total of 222 patients underwent biopsy following focal HIFU. Of these, 111 were within our three earlier phase 1/2 studies [13], [14], [15] and 111 had for-cause biopsies as a result of rising PSA and/or suspicious mpMRI after focal HIFU. Overall, 29 men had histological in-field recurrence on biopsy, while 16 had histological evidence of out-of-field de novo cancer or progression of untreated low-risk disease. A further 11 patients had both in-field and out-of-field disease on biopsy.

No patients had bleeding requiring intervention or transfusion. Within 6 mo of treatment, postoperative urinary tract infection and epididymo-orchitis occurred in 53/625 (8.5%) and 12/625 cases (1.9%), respectively. Endoscopic interventions for lower urinary tract symptoms at any time point were required in 60/625 men (9.6%). There were two (0.3%) recto-urethral fistulae. One of these cases healed following urinary diversion using urethral and suprapubic catheters for 6 mo but no bowel diversion. The other required open reconstructive surgery because of failure of conservative management (Table 4).

**Table 4:** Clavien-Dindo classification of post-HIFU complications

Clavien-Dindo grade	Complication	Incidence, *n*/*N* (%)
I	Urinary tract infection	53/625 (8.5)
I	Epididymo-orchitis	12/625 (1.9)
IIIa	Rectourethral fistula	1/625 (0.2)
IIIb	Endoscopic procedures for LUTS	60/625 (9.6)
IIIb	Rectourethral fistula	1/625 (0.2)

HIFU = high-intensity focused ultrasound; LUTS = lower urinary tract symptoms.

Baseline urinary continence status was reported by 421 men using the EPIC urinary domain questionnaire. At 1–2 and 2–3 yr after focal HIFU, pad-free status was available at both baseline and follow-up for 313 and 247 men, respectively; 304 (97%) and 241 (98%) were pad-free (0 pads) at these two time points. No men required more than 1 pad/d, so social continence was achieved in 100%. At 1–2 and 2–3 yr after focal HIFU, pad-free, leak-free status at both baseline and follow-up was available for 250 and 195 men, respectively; 208 (83%) and 156 (80%) were pad-free, leak-free continent at these two time points (Table 5).

**Table 5:** Patient-reported outcome measure for urinary incontinence according to the EPIC urinary domain among men undergoing focal HIFU for nonmetastatic prostate cancer

Patient-reported urinary incontinence	Patients, *n* (%)	
	1–2 yr FU	2–3 yr FU
0 pads	304/313 (97)	241/247 (98)
0–1 pads	313/313 (100)	247/247 (100)
No leakage at all	208/250 (83)	156/195 (80)

EPIC = Expanded Prostate Cancer Index Composite; HIFU = high-intensity focused ultrasound; FU = follow-up.

In both univariable and multivariable analyses, only pre-HIFU PSA and stage T3 were significant in relation to failure, with multivariable hazard ratios of 1.04 (95% CI 1.01–1.07; *p* = 0.004) and 3.06 (95% CI 1.11–8.44; *p* = 0.03), respectively (Supplementary Table 1). The uncorrected *c* statistic was 0.67; after correction for optimism, the value was 0.66.

## 4 Discussion

Our study shows that following focal HIFU, failure-free survival was 88% at 5 yr. Metastasis-free survival was 98% and cancer-specific survival was 100% at 5 yr. Only 2% had urinary incontinence requiring use of one daily pad and no men required more than 1 pad/d. Bowel complications were rare (0.3%).

The strengths of our study lie in its large and multicentre nature, with medium-term follow-up data prospectively collected in a nationally mandated registry and with independent quality control of data entry against source records. We predominantly treated patients with clinically significant prostate cancer, for whom it is widely accepted that treatment is required because of a higher risk of progression on active surveillance.

While it is accepted that whole-gland radical prostatectomy and radical radiotherapy are effective in treating clinically significant nonmetastatic prostate cancer, these modalities involve significant probabilities of urinary and sexual side effects, and some bowel toxicity in the case of radiotherapy. Focal therapy, by treating known areas of cancer with a margin, aims to preserve prostate tissue and minimise damage to the neurovascular bundles, bladder neck, external urethral sphincter, and rectum. We have shown that this strategy has a low probability of treatment-related side effects and achieves good cancer control in the medium term. Despite the absence of long-term follow-up, we find these results reassuring and acceptable considering that we predominantly used focal HIFU in intermediate- and high-risk cancer.

Focal therapy using various energy modalities has been evaluated in single-arm retrospective and prospective studies. Recent systematic reviews have shown that focal therapy has a minimal impact on quality of life, and while oncological effectiveness is yet to be established, genitourinary function is well preserved [8], [29].

Most of our low-risk cases were historical from a time when active surveillance for such disease was still questioned and it was deemed appropriate to offer these men focal HIFU as an alternative to radical therapy. Nonetheless, these cases still required a minimum amount of Gleason 6 cancer to qualify. Our UK focal HIFU programme now does not allow treatment in instances of low-volume Gleason 6 disease because the probability of progression for such lesions is rare, unless there are extenuating circumstances such as psychological distress to the patient due to being on active surveillance or family history [30]. This stance contrasts with some other studies that have predominantly treated very low-risk or low-risk disease with focal therapy [16], [17], [19].

While long-term data are awaited, randomised comparative studies among patients with intermediate-risk cancers are currently being piloted [31]. However, because of previous problems in maintaining physician and patient equipoise in 11 failed randomised comparative trials comparing different interventions for localised prostate cancer, this might not be possible to deliver [32]. Even if randomisation could be delivered, the sample size would have to be based on a noninferiority design that might require between 2000 and 8000 patients recruited, randomised and followed up over 10–15 yr, depending on the noninferiority margin used.

However, other treatments such as prostate brachytherapy and robotic prostatectomy have been approved for clinical use without or before the completion of randomised comparative studies. Partial nephrectomy for the treatment of renal cancer is another example. Such changes in practice were often based on medium-term outcomes from cohort studies because the long natural history of prostate cancer and small renal tumours made it unfeasible to deliver randomised controlled trials. In this context, physicians and health care organisations will need to consider whether it is justified to insist on randomised comparative data for focal therapy compared to radical therapy powered on mortality and metastases. On the basis of our data, patients currently diagnosed with prostate cancer that is suitable for focal therapy may prefer to have the option to choose whole-gland radical therapy or focal therapy.

### 4.1 Limitations

We accept that there are some limitations to our study. First, since our prospective registry was embedded in clinical care, not all patients were routinely biopsied after treatment. We validated the role of mpMRI for follow-up after focal-HIFU compared to prostate biopsy and showed that the negative predictive value for mpMRI in this setting is ≥95% for clinically significant prostate cancer [25]. Second, in light of not having a validated and accepted cancer control measure, we considered a clinically meaningful composite outcome measure that reflects the recent Intermediate Clinical Endpoint in Carcinoma of the Prostate consensus findings [33]. Finally, validated questionnaire data were not available for all patients owing to our reliance on postal return of questionnaires. While we used the International Index of Erectile Function (5-item) questionnaire, these data were unavailable at the time of analysis.

## 5 Conclusions

Focal therapy using HIFU could be offered to select patients with clinically significant nonmetastatic prostate cancer as it is effective in the medium term and has a low probability of urinary and rectal side effects.

This study was presented in May 2016 at the American Urology Association Annual meeting.

  ***Author contributions***: Hashim U. Ahmed had full access to all the data in the study and takes responsibility for the integrity of the data and the accuracy of the data analysis.  

*Study concept and design*: Ahmed, Emberton, Charman, van der Meulen.

*Acquisition of data*: Guillaumier, Charman, McCartan, Hosking-Jervis, Moore, Nigam, Virdi, Ogden, Afzal, Peters, Arya, Dudderidge, Hindley, Lewi, Persad, Shah, Winkler, Emberton, Ahmed.

*Analysis and interpretation of data*: Ahmed, Charman, Emberton, Guillaumier, van der Meulen.

*Drafting of the manuscript*: Ahmed, Guillaumier.

*Critical revision of the manuscript for important intellectual content*: Guillaumier, Ahmed, Emberton, Charman.

*Statistical analysis*: Charman, van der Meulen.

*Obtaining funding*: Ahmed, Emberton.

*Administrative, technical, or material support*: McCartan, Hosking-Jervis.

*Supervision*: Emberton, Ahmed.

*Other*: None.  

***Financial disclosures:*** Hashim U. Ahmed certifies that all conflicts of interest, including specific financial interests and relationships and affiliations relevant to the subject matter or materials discussed in the manuscript (eg, employment/affiliation, grants or funding, consultancies, honoraria, stock ownership or options, expert testimony, royalties, or patents filed, received, or pending), are the following: Hashim U. Ahmed has received research funding from the Wellcome Trust, Prostate Cancer UK, Sonacare Inc., Trod Medical, and Sophiris Biocorp; consultant fees from Sophiris Biocorp and Sonacare Inc.; and proctor fees for training surgeons in HIFU. Mark Emberton has received funding from NIHR-i4i, MRC, Sonacare Inc., Trod Medical, the Cancer Vaccine Institute and Sophiris Biocorp; consultant fees from Sonacare Inc., Sophiris Biocorp, Steba Biotech, Exact Imaging, and Profound Medical; and proctor fees for training surgeons in HIFU. He owns loan notes/stock options in Nuada Medical. Caroline M. Moore has received research funding from the National Institute for Health Research, The European Association of Urology Research Foundation, Prostate Cancer UK, Movember, and the Cancer Vaccine Institute; advisory board fees from Genomic Health; and proctor fees for training surgeons in HIFU. Richard G. Hindley has received proctor fees for training surgeons in HIFU and owns loan notes/stock options in Nuada Medical. Manit Arya has received proctor fees for training surgeons in HIFU. Mathias Winkler has received a travel grant and loan of a device from Zicom Biobot. The remaining authors have nothing to disclose.  

***Funding/Support and role of the sponsor*****:** This study was supported by a Clinician Scientist Fellowship awarded by the Medical Research Council (UK) to H.U. Ahmed (Grant G1002509) and an unrestricted grant from Sonacare Inc. The sponsors played a role in data collection.
