# Supplementary material for: A Multicentre Study of 5-year Outcomes Following Focal Therapy in Treating Clinically Significant Nonmetastatic Prostate Cancer
Source: Eur Urol. 2018 Oct;74(4):422–9. doi: 10.1016/j.eururo.2018.06.006 (PMC6156573; doi:10.1016/j.eururo.2018.06.006)
Supplement: Supplementary file 1 [file mmc1.docx]

**Supplementary Tables.**

1. Cox regression model for progression free survival based on PSA density, Gleason score and T stage. (n=538)

| **Cox regression model for progression free survival** | | | | | |
| --- | --- | --- | --- | --- | --- |
| **Univariable analysis** | **HR (95%-CI)** | **p-value** | **Multivariable analysis** | **HR (95%-CI)** | **p-value** |
| **Age (per year increase)** | 1.01 (0.98-1.05) | 0.5 | **Age (per year increase)** | NS | NS |
| **Prostate Volume (per cc increase)** | 1.01 (0.99-1.02) | 0.4 | **Prostate Volume (per cc increase)** | NS | NS |
| **Pre-HIFU PSA (per point increase)** | 1.04 (1.01-1.07) | 0.005 | **Pre-HIFU PSA (per point increase)** | 1.04 (1.01-1.07) | 0.004 |
| **Gleason Score**  7  8-10 | 1.63 (0.86-3.08)  3.57 (0.80-16.00) | 0.1  0.1 | **Gleason Score**  7  8-10 | NS | NS |
| **T Stage** 2  3 | 1.24 (0.49-3.15) 3.11 (1.13-8.56) | 0.7 0.03 | **T Stage** 2  3 | NS 3.06 (1.11-8.44) | NS 0.03 |
| Abbreviations: HR=hazard ratio, HIFU=high intensity focused ultrasound, PSA=prostate specific membrane antigen, NS=not significant.  n=22 patients were deleted in the multivariable analysis due to missing data points in the determinants. The total dataset was therefore n=577 with 60 events. There was significant no difference between the patients with and without missing data in the model. | | | | | |

2.

| **Erectile function (IIEF-5)** | Erections sufficient to maintain penetrative sexual activity | 138/165 (84%) | 87/101 (86%) |
| --- | --- | --- | --- |
